# In Vitro Evaluation of Antibacterial, Antioxidant, and Antidiabetic Activities and Glucose Uptake through 2-NBDG by Hep-2 Liver Cancer Cells Treated with Green Synthesized Silver Nanoparticles

**DOI:** 10.1155/2022/1646687

**Published:** 2022-05-17

**Authors:** Shahnaz Majeed, Mohammed Danish, Norul Aini Zakariya, Rokiah Hashim, Mohammed Tahir Ansari, Saad Alkahtani, Md Saquib Hasnain

**Affiliations:** ^1^Faculty of Pharmacy and Health, Universiti Kuala Lumpur, Royal College of Medicine, Ipoh Perak, Malaysia 30450; ^2^Bioresource Technology Section, School of Industrial Technology, Universiti Sains Malaysia, 11800 Minden, Penang, Malaysia; ^3^School of Pharmacy, Faculty of Science and Engineering, University of Nottingham Malaysia, Jalan Broga, Semenyih, 43500 Selangor, Malaysia; ^4^Department of Zoology, College of Science, King Saud University, P. O. Box 2455, Riyadh 11451, Saudi Arabia; ^5^Department of Pharmacy, Palamau Institute of Pharmacy, Chianki, Daltonganj, 822102 Jharkhand, India

## Abstract

The alarming rise in diabetes owing to drug resistance necessitates the implementation of prompt countermeasures in the treatment module of diabetes. Due to their unique physicochemical features, silver nanoparticles may have potential applications in the medical and pharmaceutical industries. Silver nanoparticles (AgNPs) were synthesized from the culture filtrate of Salmonella enterica (ATCC-14028). UV-Vis spectrophotometry, FTIR, SEM, and energy dispersive X-rays were used in the characterization of the nanoparticles. Transmission electron microscopy (TEM) revealed that AgNPs are spherical and highly scattered and vary in size from 7.18 nm to 13.24 nm. AgNP stability and protein loss were confirmed by thermogravimetric analysis (TGA) at different temperatures. The AgNPs had excellent antibacterial activity and a strong synergistic effect against methicillin-resistant bacteria Staphylococcus aureus (MRSA) ATCC-4330 and Streptococcus epidermis (MRSE) ATCC-51625. The DPPH experiment revealed that the AgNPs had high antioxidant activity. The antidiabetic assay revealed that these AgNPs had an IC50 for alpha-amylase of 428.60 *μ*g/ml and an IC50 for alpha-glucosidase of 562.02 *μ*g/ml. Flow cytometry analysis of Hep-2 cells treated with AgNPs (40 *μ*g/ml) revealed higher expression of 2-NBDG glucose absorption (uptake) compared to control metformin. These AgNPs have promising antidiabetic properties and could be used in pharmaceuticals and biomedical industries.

## 1. Introduction

The branch of nanobiotechnology deals with particles in the size range of 1 to 100 nm. Nanoparticles have been reported to be used in a variety of fields, including engineering, medicine, pharmaceuticals, and textiles [1–3]. Metallic nanoparticles, particularly platinum, silver, and gold, have been studied for a wide range of applications including antibacterial, antifungal, anticancer, catalysis, and electronics [4]. Among these nanoparticles, silver nanoparticles have recently piqued the interest of researchers due to their unique chemical and physical properties, particularly in the electrical, thermal, catalysis, industrial, and biomedical fields [5, 6]. AgNPs have potential applications in medical device coating, sensors, the ph5armaceutical industry, drug delivery, orthopaedics, and oncology [7]. It is also possible to alter the physical, biological, and chemical properties of metallic nanoparticles because they have a low surface area-to-volume ratio [8]. Study shows that the silver nanoparticles from the Hypnea valentiae exhibited outstanding antioxidant, antibacterial, and anticancer properties [9].

Diabetes mellitus is a serious metabolic disorder characterized by elevated glucose levels in the blood that can be acute or chronic [10]. Increased appetite, frequent urination, and increased thirst are the most common symptoms. Diabetes mellitus, if left untreated, can lead to serious health problems such as chronic kidney disease, stroke, cancer, neuropathy, vision loss, and cardiovascular disease [11]. Diabetes mellitus affects people in both developed and developing countries, with estimates indicating that about 25% of the world's population is affected [12]. Most of the antidiabetic drugs such as sulphonylurea, alpha-glucosidase inhibitors, biguanides, and thiazolidinediones decrease the glucose level in the body. Secretagogues like sulfonylurea depolarize the cell membrane, enabling influx of calcium ions into the beta cells and hence increasing insulin secretion from the secretory granules [13]. Although antidiabetic drugs have been used for a long time, the biggest concern with them is that diabetic patients are developing drug resistance and suffering from drug toxicity, weight gain, gastrointestinal disturbance, lactic acidosis, and, in some cases, liver disease [14, 15].

Studies have reported the use of nanotechnology in management of diabetes. The nanoparticles can be used to develop glucose sensor technology capable of measuring the accurate glucose level in the body [16, 17]. Controlled insulin delivery is possible with nanomedicine because it can detect changes in the blood glucose levels and automatically control the release of insulin to keep the blood glucose levels normal [18]. Nanoparticulate cancer drugs, such as Abraxane, Onivyde, and Doxil, may improve the efficacy of drug delivery to target sites [19, 20]. The current research will help to explore the function of AgNPs that will help in delivering the drugs to the target site by conjugating the nanoparticles with available commercial drugs, which would be more potent to produce biological efficacy.

The broad applications of AgNPs have been explored, such as catalysis, dye reduction, antimicrobial, anticancer, and household goods. As a result, it piques the interest of researchers to investigate more and more applications for synthesizing AgNPs. There are various methods for the synthesis of AgNPs, such as physical, chemical, and biological methods. Both physical and chemical methods have been found to be extremely time-consuming, expensive, and potentially harmful to the environment and human health [21, 22]. The biological method, which employs fungi, bacteria, plants, and algae as nanofactories, has been shown to be nontoxic, environmentally friendly, and cost-effective, and it has a wide range of applications in biomedical, industrial, and pharmaceutical settings [23, 24].

There is a lack of data to explain how AgNPs will improve glucose uptake in Hep-2 cells. This project is aimed at synthesizing AgNPs from Salmonella enterica and exploring its antioxidant, antidiabetic, and antibacterial effects. The synthesized AgNPs were characterized using UV-Vis spectroscopy, FTIR, TGA, and SEM/EDX/TEM and were found to be consistent with previous studies. The study also examined the effects of AgNPs on the enzymatic activity of alpha-glucosidase and alpha-amylase. Flow cytometry was used to study glucose absorption in treated Hep-2 liver cancer cells, and the antibacterial activity was carried out against MRSA and MRSE using the disc diffusion method.

## 2. Material and Methods

### 2.1. Chemical and Reagents

Silver nitrate (AgNO_3_), nutrient agar media, DMEM-low glucose, foetal bovine serum, and MTT reagent were procured from HiMedia, Malaysia. Metformin and DMSO were obtained from Sigma-Aldrich, Bangalore, India.

### 2.2. Cell Line and Bacteria Culture

The Hep-2 cell line was purchased from the National Centre of Cell Science Studies (NCCS), Pune, India, and Salmonella enterica was procured from American Type Culture Collection (ATTC).

### 2.3. Synthesis of AgNPs from Salmonella enterica

Salmonella enterica was cultured overnight in 100 mL nutrient agar broth in a 200 mL conical flask at 37°C. The following day, the culture was centrifuged for 10 minutes at 3000 rpm. The pellet was discarded, and the supernatant was transferred to another conical flask. The supernatant was used to synthesize AgNPs. 1 mM silver nitrate was added dropwise to the supernatant solution with constant stirring for 1 hour at room temperature (30°C). After adding the silver nitrate, the flask was placed on an orbital shaker for 12 hours to allow complete reduction of the silver nitrate. The colour change confirms that silver ions have been reduced to silver nanoparticles.

### 2.4. Characterization of AgNPs

An accurately measured 100 *μ*L of AgNP solution was suitably diluted with double-distilled water (dH_2_O), and the sample was scanned from 300 to 600 nm using a UV-visible spectrophotometer (Shimadzu-80712, Japan). The potassium bromide (KBR) pellet method was used for the FTIR analysis. Solid AgNPs were mixed with KBR (1 : 10) and pressed into a solid pellet, and FTIR spectra were analyzed (Shimadzu IRPRESTIGE 21, Japan). AgNP sample spectrum was collected by 64 scans aggregating from the 4000 cm^−1^ to 400 cm^−1^ wave region. The protein and other component loss at different temperatures was analyzed using thermogravimetric analysis (TGA) (Mettler Toledo, Switzerland). The sample was placed on an aluminium pan, and the analysis was performed at a heating rate of 10°C per minute. The liquid AgNP solution was dried at 60°C for 5 days, and the dried sample was subjected to surface morphological analysis using FESEM (FEI Quanta FEG 650, Thermo Fisher Scientific, Netherlands) and for the elemental nature of silver using EDX (Oxford Instrument, Model X-Max). The AgNP solution was sonicated for 5 minutes and then placed on a copper-coated grid for shape, size, and polydispersity index using TEM analysis using Libra 120 (Carl Zeiss, Germany).

### 2.5. Determination of the Antibacterial Activity of Drug-Resistant MRSA and MRSE

The disc diffusion method was used to test the antibacterial activity of biosynthesized AgNPs on nutrient agar media. A 2-hour old MRSA and MRSE bacterial culture was inoculated and swabbed on nutrient agar medium. The blank discs were placed on the nutrient agar plate and AgNPs were then impregnated at a concentration of 10, 20, 30, and 40 *μ*g/ml, respectively, and the results were compared to the positive control vancomycin. The zone of inhibition was recorded after overnight incubation at 37°C.

### 2.6. Determination of Synergistic Effect

The synergistic effect was evaluated along with the different antibiotics such as vancomycin, ceftriaxone, and gentamycin. Antibiotic discs were impregnated with 20 *μ*g/ml of AgNPs, and a zone of inhibition was recorded after 8 h of incubation. The experiment was repeated three times.

### 2.7. Evaluation of the Antioxidant Activity of AgNPs through the DPPH Assay

The antioxidant activity of AgNPs was determined using the DPPH (1,1-diphenyl-2-picrylhydrazyl) test. Varying concentrations of AgNPs, starting from 10 *μ*g/ml to 40 *μ*g/ml, were dissolved in the DPPH solution [25]. Ascorbic acid was used as a positive control. The antioxidant activity was measured by the colour shift from purple to yellow after the addition of AgNPs, which is caused by the absorption of hydrogen atoms. Absorption was recorded using a spectrophotometer, and DPPH inhibition was calculated using the formula below.

(1) $$\begin{array}{c} \text{DPPH }\left(\%\right)=\frac{\text{Ac}–\text{As}}{\text{Ac}}\times 100, \end{array}$$

where Ac is the average absorbance for the control (ascorbic acid) and As is the absorbance for the sample (AgNPs).

### 2.8. Evaluation of the Antidiabetic Activity of AgNPs

#### 2.8.1. Determination of Alpha-Amylase Enzyme Inhibition

The starch solution (0.1% *w*/*v*) was prepared by stirring 0.1 g of potato starch in 100 ml of 16 mM sodium acetate buffer. Subsequently, the enzyme solution was prepared by mixing 27.5 mg of alpha-amylase into 100 ml of double-distilled water. The colorimetric reagent was prepared by mixing sodium potassium tartrate solution and 3,5-di nitro salicylic acid solution (96 mM). Next, starch was added to the control and AgNP solution (five concentrations: 62.5, 125, 250, 500, and 1000 *μ*g/ml) and left as such so that alpha-amylase reacts at 25°C and the reaction was measured in 3 minutes. The positive control used in this study was acarbose. The maltose production was quantified by reducing 3,5-dinitro salicylic acid to 3-amino-5-nitro salicylic acid [26]. The whole reaction was detected at 540 nm using an ELISA reader. The inhibition (*I*%) of alpha-amylase was calculated using the formula below:

(2) $$\begin{array}{c} I\%=100-\frac{\text{As}-\text{Ab}}{\text{Ac}-\text{Ab}}\times 100, \end{array}$$

where *I*% is the inhibition percentage and As, Ab, and Ac are the average absorbance of the sample, blank, and control, respectively.

#### 2.8.2. Evaluation of the Alpha-Glucosidase Enzyme Inhibition

The AgNP solutions at concentrations of 62.5, 125, 250, 500, and 1000 *μ*g/ml were mixed with starch substrate solution (2% *w*/*v* maltose or sucrose) in the presence of 0.2 M Tris buffer at pH 8.0 and incubated for 5 min at 37°C. Subsequently, 1 ml of the alpha-glucosidase enzyme (1 U/ml) was added and incubated at 35°C for 40 min [26]. The reaction was terminated with the addition of 2 ml of 6 N HCl. Voglibose was used as a standard control. The colour intensity was measured at 540 nm using an ELISA reader, and alpha-glucosidase enzyme inhibition (*I*%) was calculated using the formula below:

(3) $$\begin{array}{c} I\%=100-\frac{\text{As}-\text{Ab}}{\text{Ac}-\text{Ab}}\times 100, \end{array}$$

where *I*% is the inhibition percentage and As, Ab, and Ac are the average absorbance of the sample (AgNPs), blank, and control (voglibose), respectively.

#### 2.8.3. Evaluation of the Anticancer Activity on Hep-2 Liver Cancer Cells through the MTT Assay

A total of nearly 200 *μ*l of Hep-2 cells were seeded in a 96-well plate to get a cell density of 20,000 cells per well and allowed to grow for 24 h under a carbon dioxide environment. Varying concentrations of AgNPs 10, 20, 40, 60, and 80 *μ*g/ml were added to the cells, and the whole plate was then incubated for 24 h at 37°C in an atmosphere of 5% CO_2_. After incubation, the spent media were removed and the MTT reagent to a concentration of 0.5 mg/mL of total volume was added. The entire plate was wrapped with aluminium foil to avoid light exposure and incubated for 3 minutes. The MTT reagent was removed, and 100 *μ*l of solubilization solution (DMSO) was added. The whole plate was stirred gently on a gyratory shaker that enhanced dissolution. The absorbance was recorded using an ELISA reader at a reference wavelength of 570 and 630 nm [27].

(4) $$\begin{array}{c} \%\text{Cell viability}=\left[\frac{\text{Mean abs of treatedcells}}{\text{Mean abs of untreated cells}}\right]\times 100. \end{array}$$

#### 2.8.4. Study of the Glucose Uptake of Hep-2 Cells through the Expression of 2-NBDG (2-(N-(7-Nitrobenz-2-oxa-1,3-diazol-4-yl) Amino)-2-deoxyglucose)

Hep-2 cells were cultured in 96-well plates at a density of 2 × 10^5^ cells per 2 ml and incubated at 37°C overnight under a CO_2_ atmosphere. After 24 hours, the spent media were aspirated, and the cells were added to a glucose-free culture medium containing 100 *μ*M 2-NBDG and incubated for 2 hours with AgNPs. Metformin was used as the control. After the treatment, the medium was aspirated, and the cells were washed with PBS. PBS was removed, and 200 *μ*l trypsin was added and incubated at 37°C for 3 to 4 minutes. After that, 2 ml of culture medium was added, and cells were harvested directly in 12 × 75 mm tubes. The tubes were centrifuged for 5 minutes at 25°C at 300 × *g*. The supernatant was carefully aspirated, and the pellet was resuspended with the cells in 0.5–1 ml of PBS. The entire tube was thoroughly mixed so that the cells would separate from each other. The cells were immediately analyzed by flow cytometry [28]. The fluorescence with excitation and emission at 465 nm and 540 nm, respectively, was measured in the FL1 channel used to detect FITC and utilized to examine the uptake of 2-NBDG by Hep-2 cells.

### 2.9. Statistical Analysis

Statistical evaluation of the results was analyzed through standard error means ± standard deviation; *p* < 0.05 value was accepted as statistically significant.

## 3. Result and Discussion

### 3.1. Synthesis and Characterization of AgNPs

**Figure 1:**
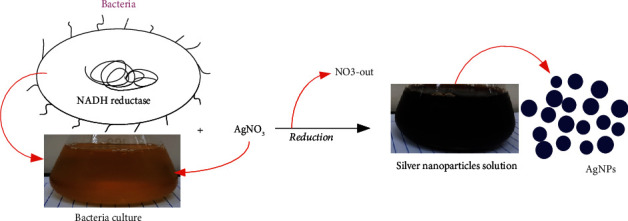
Biosynthesis of AgNP: colour change into dark brown confirms AgNP formation.

**Figure 2:**
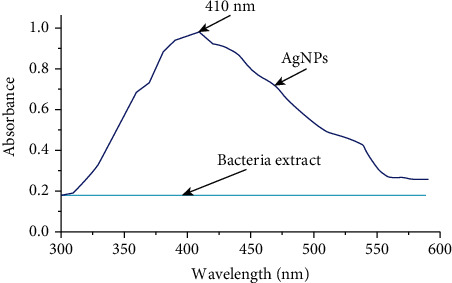
UV spectral analysis of biosynthesized AgNPs showed absorption peak at 410 nm while no peak revealed for the bacterial extract.

The reduction of silver nitrate into AgNPs occurs immediately after it comes into contact with the bacterial extract of Salmonella enterica (ATCC-14028). The dark brown solution confirms the reduction of silver nitrate (Figure 1) [29, 30]. AgNPs showed a specific absorption peak at 410 nm measured using the UV-Vis spectrophotometer, which validates the reduction of silver nitrate as shown in Figure 2 [31, 32]. Although a specific process for the reduction is unknown, some studies suggest that enzymes, such as NADH coenzyme reductase, are involved in the electron transfer (shuttle) to reduce or neutralize the Ag^+^ ions into nanoparticles [33, 34]. The biological approach used for the synthesis of various nanoparticles such as silver, gold, platinum, copper, cadmium, and zinc oxide showed excellent reduction and production of nanoparticles and contains various proteins and functional groups that stabilize the nanoparticles [35].

**Figure 3:**
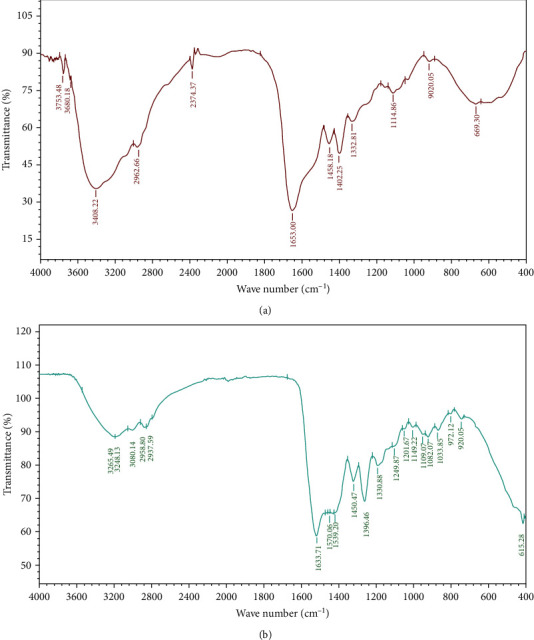
FTIR analysis of synthesized AgNPs showing that various functional groups are associated with AgNPs where (a) is FTIR of AgNPs and (b) is the FTIR for the cell-free extract.

FTIR analysis showed that various functional groups are associated with AgNPs. The prominent functional groups are 3402.22 cm^−1^ N-H stretch for amines and amides, 2962.66 cm^−1^ C-H stretch, 2374.34 cm^−1^ C-N stretch, 1653 cm^−1^ C=O amide, 1458.18 cm^−1^ C=O stretch, 1402.25 cm^−1^ C=O stretch, and 1114.86 cm^−1^ C-O primary alcohol, while FTIR for the bacterial extract showed the prominent peak at 3248.13 cm^−1^ O-H stretch for carboxylic acid, 1633.71 cm^−1^ C=C stretch alkene, 1450.47 cm^−1^ C-H bend alkane, 1396.46 cm^−1^ O-H bend phenol, 1330.88 cm^−1^ C-N stretch amine, 1082.07 cm^−1^ C-O stretch alcohol, and 1033.85 cm^−1^ CO-O-CO stretch anhydride, and also, it showed some weak peaks associated with AgNPs as shown in Figure 3. Previous studies have revealed that the nanoparticles are attached to various functional groups and biomolecules such as carbohydrates, proteins, and phenols, which help to stabilize and reduce metal ions into nanoparticles [36, 37]. The peaks at 3402 cm^−1^ and 1653 cm^−1^ are due the carbonyl stretching of the proteins [38]. The presence of these functional groups such as amines, amides, alkanes, alkenes, and carbonyl groups enhances the stability and its biological efficacy [39].

**Figure 4:**
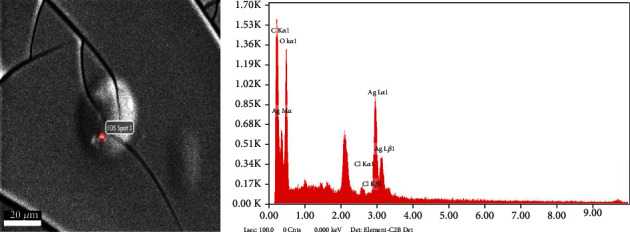
SEM analysis of AgNPs while EDX confirms the presence of element Ag.

The SEM analysis showed the surface morphology, whereas the EDX showed a prominent silver and silver purity peak (Figure 4). The morphology of the nanoparticles synthesized by the biological method differs from each other depending on reduction [40]. The SEM analysis showed that the nanoparticles are spherical in shape, while EDX showed a dominant peak of silver, which confirms the purity of silver and also confirms the presence of carbon and oxygen. The strong peak of silver is due to the absorption of silver, which corresponds to the plasmon surface resonance. Similar findings have been reported in the previous studies [41].

**Figure 5:**
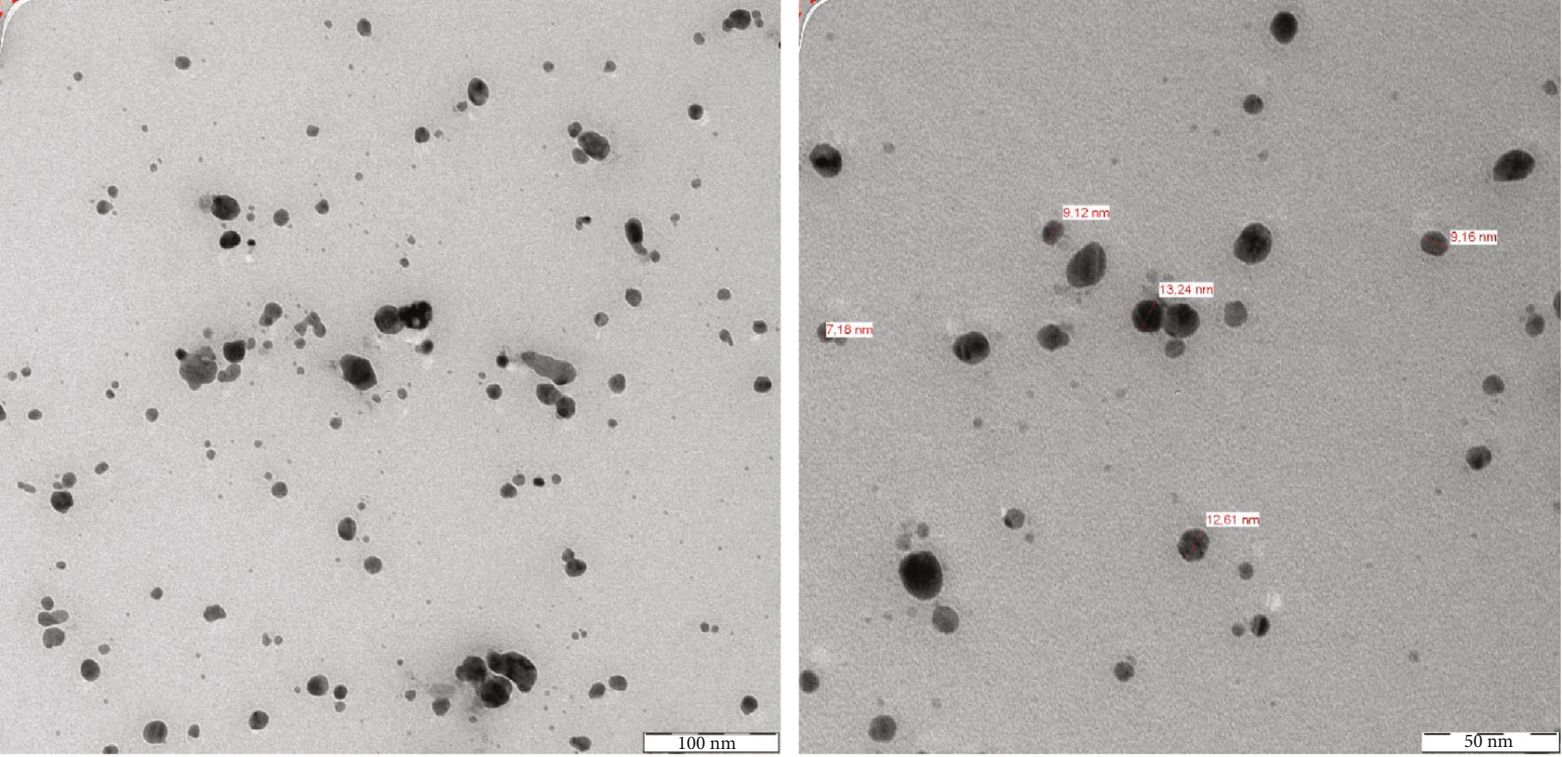
TEM analysis of synthesized AgNPs showing that particles are spherical in shape and well dispersed and their size ranges from 7.18 nm to 13.24 nm.

TEM confirms that the AgNPs are spherical in shape and are well dispersed without any agglomeration. Analysis of the TEM results confirmed that AgNPs are stable and their size ranges from 7.18 to 13.24 nm (Figure 5). Studies have shown that the shape and size of the nanoparticles have a significant impact on the antimicrobial action. The smaller the size of nanoparticles, the higher the efficacy [42]. When exposed for 30 minutes to sunlight LED (light-emitting diode), the AgNPs produced from the Dryopteris crassirhizoma rhizome extract also produced nanoparticles with sizes ranging from 5 to 60 nm. [43]. Our findings coincided with similar finding that biosynthesized AgNPs are spherical in shape, with no agglomeration [44]. The dispersion property of nanoparticles varies according to their shape, composition, and size [43].

**Figure 6:**
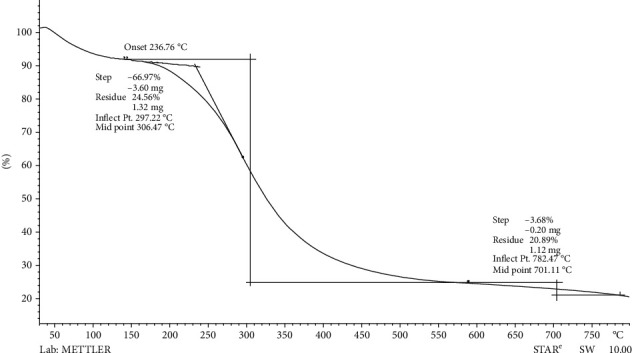
TGA of synthesized AgNPs showed the thermostability of AgNPs.

Thermogravimetric analysis (TGA) confirmed the thermal stability, volatility, moisture, and oxidation content. Thermogram was used to measure the temperature of the dried AgNPs, which ranged from 30°C to 800°C. There was no significant loss of content at 100°C. The major protein loss occurs at 236°C due to volatilization as shown in Figure 6. According to a previous study, the protein's thermal breakdown response ranges from 178°C to 288°C, depending on the purity and origin of the molecules [45]. At 782°C, the weight loss was 3.62 percent, with a final residual of 20.89 percent of the initial mass of AgNPs remaining in the sample pan, indicating that AgNPs are stable [46].

### 3.2. Antibacterial Effect of the AgNPs against MRSA and MRSE

**Figure 7:**
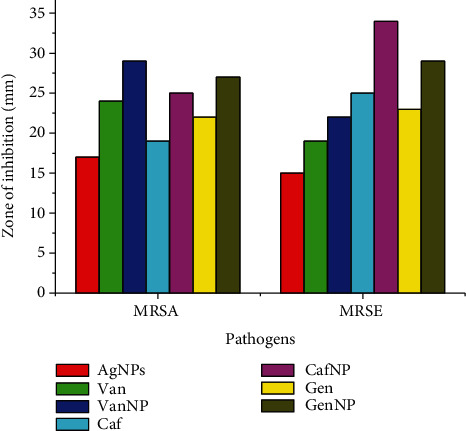
Antibacterial activity of AgNPs against MRSA and MRSE showed that AgNPs possess good antibacterial activity as well as synergistic effect against various antibiotics. Van: vancomycin; VanNP: Van+AgNPs; Caf: ceftriaxone; CafNP: ceftriaxone+AgNPs; Gen: gentamycin; GenNP: gentamycin+AgNPs.

The AgNPs were tested for their antibacterial effects against MRSA and MRSE. These AgNPs showed a minimum zone of inhibition at 10 *μ*g/ml and a maximum zone of inhibition at 40 *μ*g/ml against MRSA and MRSE. At a concentration of 40 *μ*g/ml, the zone of inhibition of 17 mm was measured against MRSA, while the zone of inhibition for MRSE was 15 mm. For testing the synergistic effect against MRSA and MRSE, 20 *μ*g/ml of AgNPs was impregnated on vancomycin (30 *μ*g/disc), ceftriaxone (30 *μ*g/disc), and gentamycin (30 *μ*g/disc). Our study showed that vancomycin combined with AgNPs demonstrated a good zone of inhibition (29 mm) against MRSA, followed by gentamycin and ceftriaxone. Ceftriaxone, on the other hand, had a larger inhibition zone (34 mm), followed by Vancomycin and gentamycine for MRSE (Table 1 and Figure 7). The experiment was repeated thrice, and an average mean was calculated. Our findings support the previous research that silver nanoparticles have potent antibacterial properties against MRSA and MRSE [47].

The AgNP binds to the bacterial cell wall and produces changes in the membrane integrity hence disrupting the bacterial cell membrane. The disruption is owed to the interaction of AgNPs with the negative charge of the cell wall. The membrane disruption may lead to cell death [47]. Additionally, when the nanoparticles penetrate the cell, they generate oxidative stress, resulting in the production of reactive oxygen species (ROS), which causes mitochondrial damage, DNA intercalation, enzyme inhibition, and lipid peroxidation [48–51].

### 3.3. Antioxidant Activity of the AgNPs

**Figure 8:**
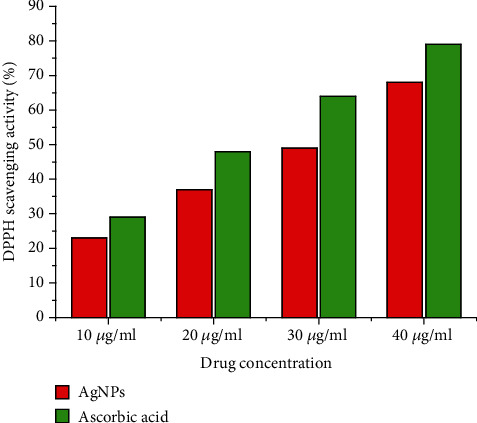
Antioxidant activity of synthesized AgNPs showed excellent antioxidant property.

The antioxidant activity of AgNPs was tested using the DPPH assay. The colour of the DPPH solution should change from purple to pale yellow, to confirm the antioxidant activity. This is due to the reduction of the DPPH radical via hydrogen atom transfer resulting in a pale-yellow solution [52]. Serially diluted solutions of 10, 20, 30, and 40 *μ*g/ml of AgNPs were used for the DPPH assay. The absorbance was measured using the UV-Vis spectrophotometer, and ascorbic acid was used as a positive control. Our findings showed that AgNPs possess good antioxidant activity, and an increase in the concentration of AgNPs increases the antioxidant activity (Figure 8). Our findings are consistent with the previous research that reported AgNPs produced from plant extract had good radical scavenging capability [53].

### 3.4. Antidiabetic Activity

#### 3.4.1. Inhibition of the Alpha-Amylase and Alpha-Glucosidase

**Figure 9:**
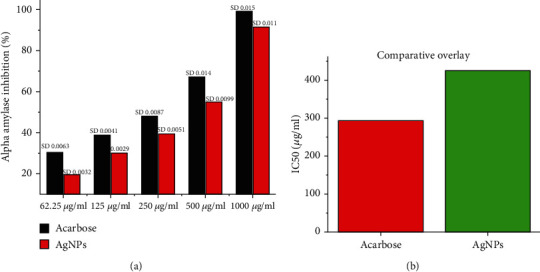
Alpha-amylase inhibition of AgNPs in a dose-dependent manner along positive control acarbose (a) while (b) showed IC50 of AgNPs and acarbose showing AgNPs able to inhibit the alpha-amylase efficiently. The readings are shown as triplicates.

The antidiabetic effect of biosynthesized AgNPs was assessed by measuring the activity of alpha-amylase and alpha-glucosidase. It was observed that AgNPs showed effective alpha-amylase inhibition at a concentration ranging from 62.5 to 1000 *μ*g/ml. These AgNPs showed an IC50 at 428.60 *μ*g/ml, compared to positive control acarbose which exhibited the IC50 at 295.42 *μ*g/ml (Figures 9(a) and 9(b)). An earlier study demonstrated that silver nanoparticles synthesized from the plant Pterocarpus marsupium effectively inhibited the activity of the enzyme alpha-amylase [54].

**Figure 10:**
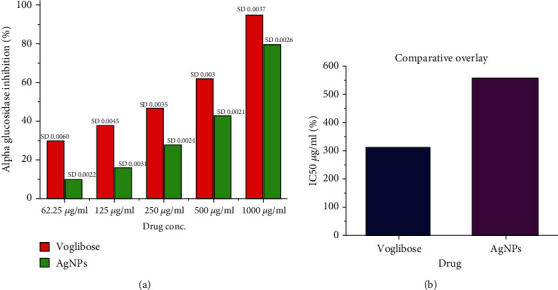
Alpha-glucosidase inhibition of AgNPs in a dose-dependent manner along with positive control voglibose (a) while (b) showed IC50 of AgNPs and voglibose. The AgNPs were able to inhibit the alpha-glucosidase efficiently. The readings are shown as triplicates.

AgNPs successfully suppressed alpha-glucosidase in a dose-dependent manner in the range of 62.5 to 1000 *μ*g/ml. The statistical data through the ELISA plate reader revealed that AgNPs effectively inhibited alpha-glucosidase in a dose-dependent manner. The IC50 of AgNPs was 562.02 *μ*g/ml, which was compared to the positive control voglibose (313.62 *μ*g/ml) as shown in Figures 10(a) and 10(b).

Silver nanoparticles synthesized from grape pomace waste showed excellent inhibition of the alpha-amylase and alpha-glucosidase enzymes [55]. Silver nanoparticles synthesized from lemongrass also exhibited antidiabetic properties which were attributed to the inhibition of alpha-amylase in a dose-dependent manner [56]. Our results are supported with comparable findings in the published data [57].

#### 3.4.2. Toxicity of the AgNPs on Hep-2 Liver Cancer Cells

**Figure 11:**
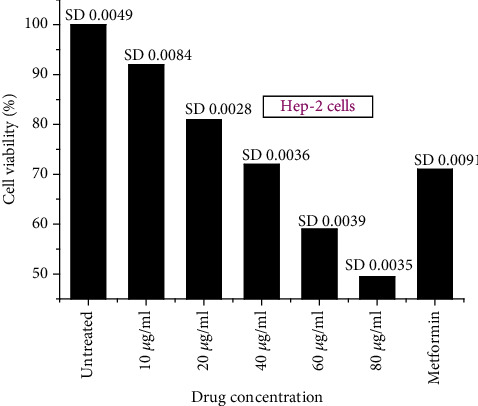
Graphical representation showing the anticancer effect of AgNPs against Hep-2 liver cancer cells in a dose-dependent manner. The readings are shown as triplicates.

**Figure 12:**
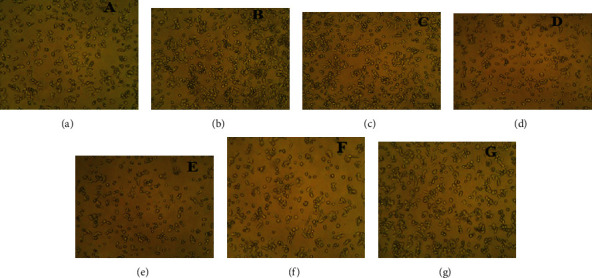
Anticancer activity of AgNPs on Hep-2 cells in a dose-dependent manner: (a) metformin; (b) cells at 10 *μ*g/ml; (c) cells at 20 *μ*g/ml and 40 *μ*g/ml; (d) cells at 40 *μ*g/ml; (e) cells at 60 *μ*g/ml; (f) cells at 80 *μ*g/ml; (g) untreated cells.

The toxicity of AgNPs was carried out in Hep-2 liver cancer cells in a dose-dependent manner. The AgNP concentration varied as 10 *μ*g/ml, 20 *μ*g/ml, 40 *μ*g/ml, 60 *μ*g/ml, and 80 *μ*g/ml, with positive control metformin at 100 *μ*M, respectively. The biosynthesized AgNPs showed the IC50 at 80 *μ*g/ml while at 40 *μ*g/ml, more than 70% of the cells were alive and compared with the cells treated with metformin (Figures 11 and 12). Silver has shown excellent anticancer activity against various cancer cells such as MDA-MB-231 breast cancer cells, U251 glioblastoma cells, and IMR-90 lung fibroblasts in a dose-dependent manner [58–60]. These AgNPs showed excellent anticancer effects and can be a probable drug candidate for the anticancer drug delivery system [61]. These AgNPs produce ROS from oxidative stress depending on the size of the nanoparticles. The smaller the particle size, the higher the production of ROS [62]. AgNPs also induce mitochondrial disruption by leaking superoxide anion and reacting with the SH group of proteins and glutathione, resulting in oxidative stress [63, 64].

#### 3.4.3. Study of the Glucose (2-NBDG) Uptake by Hep-G2 Cells Treated with AgNPs through Flow Cytometry

**Figure 13:**
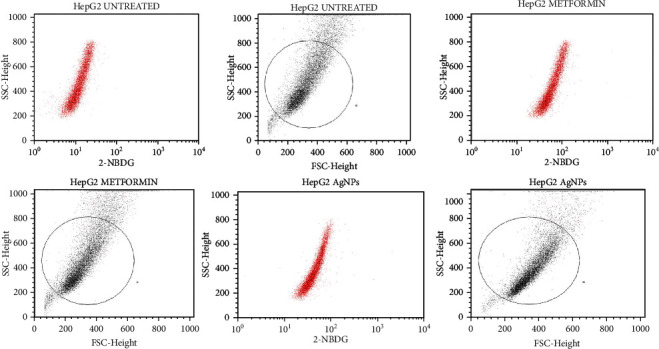
Comparative glucose uptake of the Hep-2 cells treated with AgNPs and the expression of FSC-H signal through fluorescence microscopy showing that the treated cells with AgNPs increase the uptake of glucose significantly as compared with nontreated cells.

**Figure 14:**
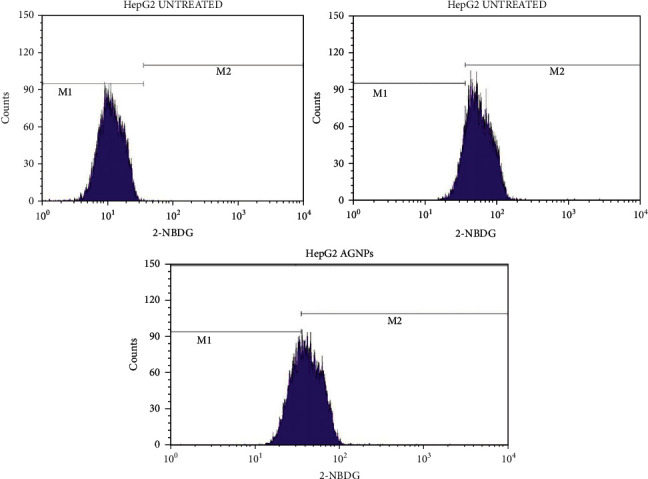
Glucose uptake study of AgNPs against HepG2 cells using BD FACSCalibur. 2-NBDG histogram of the gated HepG2 singlets distinguishes cells at the M1 and M2 phases (here M1 refers to the negative expression/region and M2 refers to the positive expression/region). Gating of M1 and M2 phases is approximate and can be refined using software (CellQuest Pro Software, version 6.0) analysis.

**Figure 15:**
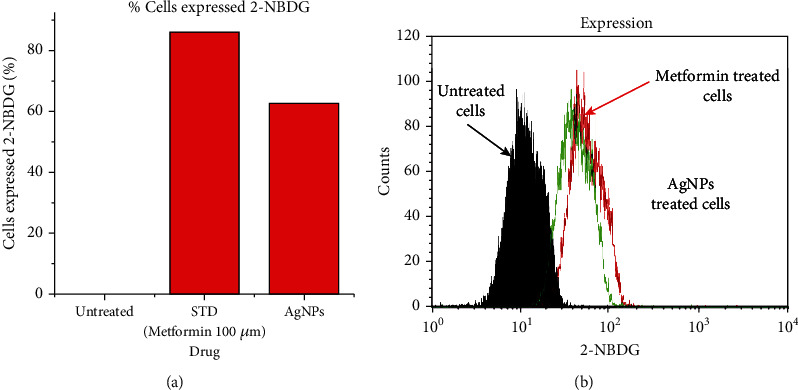
(a) Graphical representation of 2-NBDG expression. (b) Overlaid 2-NBDG expression of HepG2 cells in treated and untreated conditions.

The glucose uptake of Hep-2 cells was assessed using 2-NBDG (2-(N-(7-nitrobenz-2-oxa-1,3-diazol-4-yl) amino)-2-deoxyglucose)), a fluorescent deoxyglucose analog, and a fluorescent agent. Hep-2 cells were in glucose (2-NBDG) medium and treated with AgNPs 40 *μ*g/ml (above 70% of cells are alive) along with a positive control, 100 *μ*M of metformin (above 70% of cells are alive). Our results clearly showed that AgNP-treated Hep-2 cells enhanced glucose uptake by 62.3% while positive control showed 85.74% glucose uptake and untreated cells showed 0.12% through the expression of 2-NBDG by flow cytometry, as shown in Figures 13–15. Previous studies suggest that silver nanoparticles have good antidiabetic activity [65]. Silver nanoparticles synthesized from Halymenia poryphyroides possess excellent antidiabetic activity [66]. Herbal-mediated SNPs (HMSNPs) are nontoxic and safe and contain excellent antidiabetic properties at either a high or low concentration [67]. Silver nanoparticles synthesized from Tephrosia tinctoria possess good antidiabetic activity and improved glucose uptake in diabetic rats [68]. According to our findings, AgNPs stimulated uptake of glucose in Hep-2 cells, implying that they have excellent therapeutic and potential antidiabetic properties.

## 4. Conclusion

AgNPs produced by Salmonella enterica could be used as a reducing agent. The synthesized AgNPs are well dispersed, have limited or no agglomeration, and range in size from 7.18 nm to 13.24 nm. These AgNPs showed good synergistic antibacterial effects against MRSA and MRSE. In addition, the DPPH assay revealed that these AgNPs have significant antioxidant properties. These AgNPs were able to inhibit the alpha-amylase and alpha-glucosidase enzymes. Furthermore, these nanoparticles showed good toxicity towards Hep-2 cells. On the other hand, these AgNPs increase the absorption (uptake) of glucose treated (NBDG) in Hep-2 cells. We conclude that AgNPs synthesized from the bacterial extract exhibit excellent antibacterial, antioxidant, and antidiabetic activity and thus have the potential to be used in therapeutic applications. However, in vitro and in vivo research is needed considerably towards the toxicity and efficacy of these AgNPs on healthy human cells, which needs to be explored before using them as a strong therapeutic agent.

## Figures and Tables

**Table 1:** Antibacterial activity of AgNPs on MRSA and MRSE and their synergistic effect against various antibiotics. Van: vancomycin; Van+ NP: vancomycin plus AgNPs; Caf: ceftriaxone; Caf+NP: ceftriaxone plus AgNPs; Gent: gentamycin; Gent+ NP: gentamycin plus AgNPs. Experiment repeated three times and average value.

Bacteria	AgNPs	Van	Van+NP	Caf Caf+NP	Gen	Gen+NP
MRSA	17 ± 0.33	24 ± 1.22	29 ± 0.83	19 ± 0.5525 ± 0.66	22 ± 1.33	27 ± 0.69
MRSE	15 ± 1.22	19 ± 0.83	22 ± 0.23	25 ± 0.6634 ± 0.88	23 ± 0.45	29 ± 1.33

## Data Availability

All the data used to support the findings of this study are included within the article.
